# Automated assessment of brain MRIs in multiple sclerosis patients significantly reduces reading time

**DOI:** 10.1007/s00234-024-03497-7

**Published:** 2024-11-08

**Authors:** Victoria Sieber, Thilo Rusche, Shan Yang, Bram Stieltjes, Urs Fischer, Stefano Trebeschi, Philippe Cattin, Dan Linh Nguyen-Kim, Marios-Nikos Psychogios, Johanna M. Lieb, Peter B. Sporns

**Affiliations:** 1 Department of Neuroradiology, University Hospital Basel, Basel, Switzerland; 2 Department of Radiology and Nuclear Medicine, University Hospital Basel, Basel, Switzerland; 3 Department of Neurology, University Hospital Basel, Basel, Switzerland; 4 Department of Radiology, Netherlands Cancer Institute, Amsterdam, The Netherlands; 5 Department of Biomedical Engineering, University of Basel, Allschwil, Switzerland; 6 Department of Radiology, Neuroradiology and Nuclear Medicine, Stadtspital Zürich, Zürich, Switzerland; 7 Department of Diagnostic and Interventional Neuroradiology, University Medical Center Hamburg-Eppendorf, Hamburg, Germany; 8 GROW School for Oncology and Reproduction, Maastricht University, Maastricht, The Netherlands

**Keywords:** Multiple sclerosis, MRI, Magnetic resonance imaging, Automated assessment, AI, Artificial intelligence

## Abstract

**Introduction:**

Assessment of multiple sclerosis (MS) lesions on magnetic resonance imaging (MRI) is tedious, time-consuming, and error-prone. We evaluate whether assessment of new, expanding, and contrast-enhancing MS lesions can be done more time-efficiently by radiologists with assistance of artificial intelligence (AI).

**Methods:**

Baseline and three follow-up (FU) MRIs of thirty-five consecutive patients diagnosed with MS were assessed by a radiologist manually, and with assistance of an AI-tool. Results were discussed with a consultant neuroradiologist and time metrics were evaluated.

**Results:**

The mean reading time for the resident radiologist was 9.05 min (95CI: 6.85–11:25). With AI-assistance, the reading time was reduced by 2.83 min (95CI: 3.28–2.41, *p* < 0.001). The reading decreased steadily from baseline to FU3 for the resident radiologist (9.85 min baseline, 9.21 FU1, 8.64 FU2 and 8.44 FU3, *p* < 0.001). Assistance of AI further remarkably decreased reading times during follow-ups (3.29 min FU1, 3.92 FU2, 3.79 FU3, *p* < 0.001) but not at baseline (0.26 min, *p* = 0.96). The *baseline* reading time of the resident radiologist was 5.04 min (*p* < 0.001), with each lesion adding 0.14 min (*p* < 0.001). There was a substantial decrease in the *baseline* reading time from 5.04 min to 1.59 min (*p* = 0.23) with AI-assistance. Discussion of the reading results of the resident with the neuroradiology consultant (as usual in clinical routine) was exemplary done for FU-3 MRIs and added another 3 min (CI:2.27–3.76) to the reading time without AI-assistance.

**Conclusion:**

We found that AI-assisted reading of MRIs of patients with MS may be faster than evaluating these MRIs without AI-assistance.

## Introduction

Multiple sclerosis (MS) is the leading neuroinflammatory disease in Europe and is associated with high morbidity, long-term disability and socioeconomic burden [1, 2]. Magnetic resonance imaging (MRI) is a mainstay for the diagnostic work-up of patients with (suspected) MS [3]. By visualizing demyelinating and neurodegenerative processes in the central nervous system, MRI represents the key tool for diagnosis and monitoring of disease course and therapy in MS patients [4]. One of the major challenges in using MRI for MS is the segmentation of areas of demyelination, whose number, location and appearance are key for a tailored treatment [4, 5].

A main focus is the delineation of new MS lesions on T2/FLAIR-sequences occurring between two consecutive exams. It is known, that there is a direct link between accumulation of new lesions and increasing handicap [6] and a whole spectrum of Disease Modifying Drugs is approved for MS therapy. To date, a large number of techniques have been proposed for the automatic segmentation of MS lesions in Flair-sequences [7–9]. Automating the detection of these new lesions or helping neuroradiologists to identify them would therefore be a major advance for evaluating the patient disease progression and response to treatment.

Further, contrast-enhancing (CE) lesions, usually assessed on post-contrast T1-weighted sequences, point towards acute demyelinating processes, and have important implications for first-time diagnosis and changes in the therapeutic regime [10]. Contrast enhancement characterizes lesions that are typically not older than eight weeks and is the key surrogate marker for active inflammation. The detection of contrast-enhancing lesions next to non-enhancing lesions proves that demyelination has occurred at multiple time points, and is one important diagnostic criterion for MS [4, 5]. Contrast enhancement is associated with the occurrence of clinical relapses and the amount or volume of CE lesions is highly relevant for the evaluation of treatment efficacy [11]. Thus, an accurate and robust detection of CE lesions is critical for clinical decision making in MS patients.

Although CE lesions are an important finding on brain MRI of patients with MS, they may be easily missed by the radiologist. The morphology of CE lesions differs substantially between patients and scans with respect to size, shape (ring, punctual or linear enhancement), intensity and location [12]. Due to the steadily increasing patient throughput and the growing amount of imaging data, qualitative assessment of conventional MRI sequences comes along with non-negligible intra- and inter-observer variability having relevant implications for subsequent treatment decisions [8, 13]. Manual segmentation of CE lesions for further research or clinical questions remains a very time-consuming, tedious and error-prone task, which can become practically infeasible in clinical routine when dealing with large amounts of data under time constraints [8, 13]. Consequently, fully automated detection and segmentation of CE lesions is highly desirable and might have an impact on the diagnostic workup of MS patients.

However, in order to be used in clinical routine, several steps need to be completed. One important step to convince neuroradiologists to use automated solutions are the actual time savings for analyzing MRIs, when using AI-based decision support.

Therefore, we hypothesized that reading of MRIs of patients with MS will be faster if the neuroradiologist is supported by Ai-based decision support compared to reading by a neuroradiologist alone.

## Methods

### Study design

This is a retrospective analysis of 35 consecutive patients diagnosed with MS who underwent baseline MRI between April 2018 and December 2020 and had at least 3 follow-up (FU) - MRIs available. MRIs included 3D-Flair sequences and 3D-T1- sequences after administration of intravenous gadolinium-based contrast. All patients underwent MRIs using Siemens scanners with 1.5 Tesla (Siemens MAGNETOM Avanto FIT 1.5T, Siemens Healthcare, Erlangen, Germany) and 3 Tesla (Siemens MAGNETOM Skyra FIT 3T, Siemens Healthcare, Erlangen, Germany) field strengths.

The study was approved by the local ethics committee. All study protocols and procedures were conducted in accordance with the Declaration of Helsinki.

The data that support the findings of this study are available from the corresponding author upon reasonable request.

### Image analysis

All MRI datasets were exported to a research server (NORA Imaging Platform, Freiburg, Germany [14]).

Manual assessment of MRIs was done by a radiology resident (4 years’ experience) and a neuroradiologist (11 years). Results of the reading of the resident were discussed with a consultant as this is the usual process in clinical routine. The time that the resident radiologist and the neuroradiologist needed for assessment of the MRIs was recorded. The time of discussion between the resident and consultant was added, as this represents the usual clinical routine.

All FLAIR sequences of the baseline MRI and 3 follow-up MRIs were assessed for lesions in the following regions: cortical, juxtacortical, periventricular, infratentorial. Additionally, the total number of lesions was calculated and MRIs were rated for new and progressive lesions.

Post-contrast T1-weighted sequences were evaluated for enhancing lesions.

This was done once by the reader alone and once with decision support provided by the automated tool.

### Automated tool

The computational pipeline begins with preprocessing of both T1 and FLAIR images using N4 bias field correction [15]. The FLAIR images are then affine co-registered and transformed using ELASTIX [16].

Automatic lesion segmentation is performed using the multi-dimensional gated recurrent units (MD-GRU) algorithm [17], trained on data from 785 patients from the Swiss Multiple Sclerosis Cohort (SMSC).

To locate lesions in space according to the McDonald diagnostic criteria, we employ FreeSurfer [18] and additional post-processing to segment various neuroanatomical structures. Furthermore, we utilize the Hammers Atlas, which includes 95 regions, and condense the subregions into 23 key areas such as the temporal lobe, cingulum, basal ganglia ect. This atlas, in MNI-space, is inversely transformed to T1 space using Dartel from SPM12 [19].

In follow-up studies, images are affine co-registered to the last study. After subtracting the lesion map of both studies, standalone lesions are identified as new lesions, while lesions attached to old ones are defined as progression.

For MS contrast agent enhancement, the script applies a median filter, generates a mask, calculates thresholds, labels connected components, and enhances potential lesions. Vessel images are identified if their intensity exceeds 600, and any overlapping lesions are excluded.

Finally, T1 and FLAIR images, along with the lesion map, ROIs following McDonald diagnostic criteria, and the Hammers Atlas, are visualized on NORA. Lesions and ROIs can be edited if necessary. Based on overlap with ROIs or the atlas, a live report is generated as a table for each study.

### Statistical analysis

To account for the longitudinal nature of the data, with multiple measurements over time for the same patient, we employed linear model with mixed effects. Factors accounted for in the analysis were the patient and the timepoint, coded as baseline, follow-up 1, follow-up 2 and follow-up 3. All reading times were converted as decimal minutes. Statistical testing was performed via ANOVA. For comparing reading times at baseline between neuroradiologist with and without AI-assistance, as all the samples were independent from each other, a paired t-test was used. 95% Confidence intervals were computed via the standard error. The analysis was implemented in R (version 4.2.1, 2022-06-23).

## Results

**Fig. 1:**
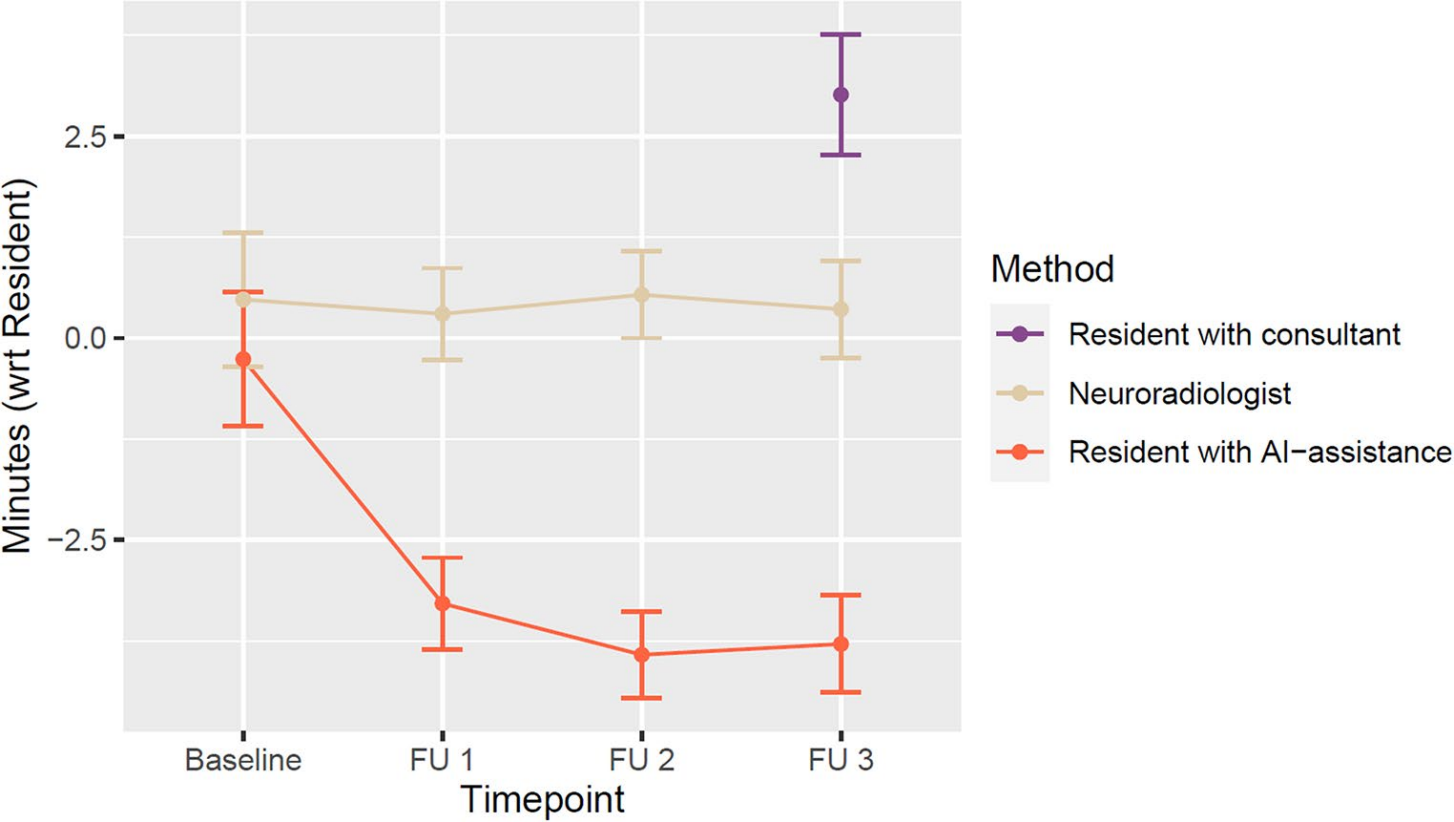
Comparative reading times with respect to the resident radiologist. Reading time for the consultant was available only for FU3, modelled separately

The overall mean reading time for the resident radiologist, corrected for patient and timepoint effects, was 9.05 min (95CI: 6.85–11:25). With AI-assistance, the reading time was reduced by 2.83 min (95CI: 3.28–2.41, *p* < 0.001). Analyzing further the variance at each timepoint, we see a steady decrease of the reading time from baseline to FU3 for the resident radiologist (9.85 min at baseline, 9.21 at FU1, 8.64 at FU2 and 8.44 FU3, *p* < 0.001). The assistance of the AI decreased remarkably the reading times during the follow-ups (3.29 min at FU1, 3.92 at FU2, 3.79 at FU3, *p* < 0.001) but not at baseline (0.26 min decrease, *paired t-testp* = 0.96) (Fig. 1).

Finally, analyzing the variance of the disease burden, we observe a *baseline* reading time of the resident radiologist of 5.04 min (*p* < 0.001), with each lesion adding 0.14 min to the reading (*p* < 0.001), and each ml of lesions adding 0.17 min (*p* = 0.03). If we were to look at the variance of to the disease burden with AI-assistance, we observe a substantial decrease in the *baseline* reading time from 5.04 min to 1.59 min (*p* = 0.23), with similar time added per lesion (0.12 min per lesion, 0.35 min per ml of lesion, *p* < 0.001), plus the added time of the accuracy of the tool, where every misclassification of a lesion (either false positive or false negative) adds an extra 0.24 min to the reading (*p* = 0.007) (Fig. 1).

**Fig. 2:**
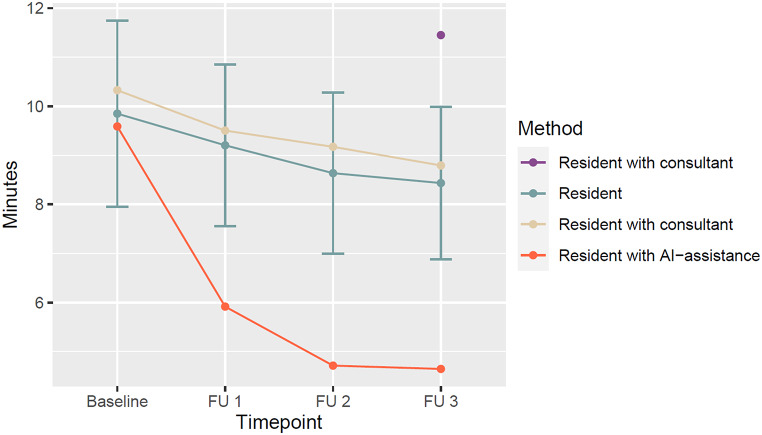
Absolute reading times per timepoint, estimated by setting the reading time of the resident as reference, and adding the estimates of other readers to it. Confidence intervals of each observer are omitted for clarity

Of note, the reading time of the neuroradiologist for the timepoints were comparable to slightly longer than of the radiology resident by 0.48 min for the baseline MRI, 0.30 (CI: -0.26–0.86), 0.54 (CI: 0.01–1.07) and 0.36 (CI: -0.24-0.96) mins for timepoints FU-1, FU-2 and FU-3, respectively. Reading times were also similar when the neuroradiologist was supported by the AI-tool with the baseline reading time being 0.20 min longer for the baseline MRI, 0.17 (CI: -0.15–0.43), 0.24 (CI: 0.11–0.46) and 0.12 (CI: -0.24-0.47) mins for the timepoints, respectively (See Fig 2).

The discussion of the reading results of the resident with the neuroradiology consultant (as usual in clinical routine) was exemplary done for FU-3 MRIs and added another 3 min (CI: 2.27–3.76) to the reading time without the AI-assisted tool.

## Discussion

Our results demonstrate that AI-assisted reading of MRIs of patients with MS is faster than evaluating these MRIs without AI-assistance. This was especially significant for follow-up examinations, which intuitively makes sense, considering that adjustments of the AI-tool are primarily performed at baseline exam, whereas all changes to this baseline can be easily and more rapidly detected with automated decision-support.

The AI-tool used for this study was trained to evaluate FLAIR on 3D-sequences. That these tasks can be performed by machine-learning tool reliably has been shown before [7–9].

High skepticism regarding applicability in the actual clinical setting remains [20, 21], although AI frameworks have been validated in their respective internal test setting, and generalizability and benchmarking were also assessed in large computational challenges such as the “WMH Segmentation Challenge 2017” (https://wmh.isi.uu.nl). In the end, a proper external validation of AI systems in clinical routine is still rarely performed. Out study therefore provides important evidence that even radiologists and neuroradiologists can actually save precious time, when using AI-based decision support. In times of increasing workloads these time-savings can directly be invested in other workflows, where AI-support is not yet available.

Another reason why the finding that the use of an AI-tool fastens reading time of neuroradiologists is important, is that only tools that make clinical workflows easier will finally be used in clinical routine. In this context, the mean time saved by AI-assistance of more than 3 min for every follow-up MRI is a huge gain in times of increasing use of imaging and greatly increasing workloads per radiologist. The additional time of around 3 min that add to the manual reading of the radiology resident reflect the usual practice in the clinical setting and even more pronounce the potential time gain of using AI-assistance.

In this study, we proposed a pipeline that utilizes NORA as a user interface for viewing and editing data. However, in order to be used in clinical routine, the ideal approach would be to integrate all these capabilities into the radiology reporting and archiving system (PACS). This would eliminate the need for radiologists to switch to third-party software. The results could be directly integrated into the final report in the form of a table. Furthermore, several steps need to be completed, such as the integration of computerized solutions in the hospital information flow and the quantification of the uncertainty associated to the automatic lesion detection, in place of the standard binary output, to leverage the clinician’s work for obvious lesion and requiring his expertise only for difficult cases. This will lead to the design of a new family of computerized medical assistants for care improvement.

This work has some limitations. First, it relies on a relatively small dataset of retrospective examinations, which were all acquired on scanner of the same vendor (Siemens). Thus, it has to be shown that the results can be generalized to other scanner types. Second, scanners included field strengths of 1.5T and 3T which may influence the performance of (neuro-) radiologists and AI-tool. Third, discussion of the reading results was only exemplary done for the third FU MRI, and may be different for other timepoints. However, the actual time savings of more than 3 min in average for every follow-up examination were large and can likely be reproduced in prospective multicenter settings.

## Conclusion

We found that AI-assisted reading of MRIs of patients with MS may be faster than evaluating these MRIs without AI-assistance. This was especially significant for follow-up examinations and may be another reason to change future practices with regard to increasing workloads of neuroradiologists.

## Data Availability

The data that supports the findings of this study is available from the corresponding author upon reasonable request.

## References

[1] Pugliatti M et al The epidemiology of multiple sclerosis in Europe. 10.1111/j.1468-1331.2006.01342.x 10.1111/j.1468-1331.2006.01342.x 16834700

[2] Wallin MT et al (2019) Global, regional, and national burden of multiple sclerosis 1990–2016: a systematic analysis for the global burden of Disease Study 2016. Lancet Neurol 18:269 30679040 10.1016/S1474-4422(18)30443-5 PMC6372756

[3] Wattjes MP et al (2021) 2021 MAGNIMS–CMSC–NAIMS consensus recommendations on the use of MRI in patients with multiple sclerosis. Lancet Neurol 20:653–670 34139157 10.1016/S1474-4422(21)00095-8

[4] Thompson AJ et al (2018) Diagnosis of multiple sclerosis: 2017 revisions of the McDonald criteria. Lancet Neurol 17:162–173 29275977 10.1016/S1474-4422(17)30470-2

[5] McDonald WI et al (2001) Recommended diagnostic criteria for multiple sclerosis: guidelines from the international panel on the diagnosis of multiple sclerosis. Ann Neurol 50:121–127 11456302 10.1002/ana.1032

[6] Sormani MP, Bruzzi P (2013) MRI lesions as a surrogate for relapses in multiple sclerosis: a meta-analysis of randomised trials. Lancet Neurol 12:669–676 23743084 10.1016/S1474-4422(13)70103-0

[7] García-Lorenzo D, Francis S, Narayanan S, Arnold DL, Collins DL (2013) Review of automatic segmentation methods of multiple sclerosis white matter lesions on conventional magnetic resonance imaging. Med Image Anal 17:1–18 23084503 10.1016/j.media.2012.09.004

[8] Danelakis A, Theoharis T, Verganelakis DA (2018) Survey of automated multiple sclerosis lesion segmentation techniques on magnetic resonance imaging. Comput Med Imaging Graph 70:83–100 30326367 10.1016/j.compmedimag.2018.10.002

[9] Bonacchi R, Filippi M, Rocca MA (2022) Role of artificial intelligence in MS clinical practice. NeuroImage Clin 35 10.1016/j.nicl.2022.103065 PMC9163993 35661470

[10] Narayana PA et al (2020) Deep learning for predicting enhancing lesions in multiple sclerosis from noncontrast MRI. Radiology 294:398–404 31845845 10.1148/radiol.2019191061 PMC6980901

[11] Barkhof F et al (2005) Predicting gadolinium, enhancement status in MS patients eligible for randomized clinical trials. Neurology 65:1447–1454 16275834 10.1212/01.wnl.0000183149.87975.32

[12] Gaj S, Ontaneda D, Nakamura K (2021) Automatic segmentation of gadolinium-enhancing lesions in multiple sclerosis using deep learning from clinical MRI. PLoS ONE 16:e0255939 34469432 10.1371/journal.pone.0255939 PMC8409666

[13] Coronado I, Gabr RE, Narayana PA (2020) Deep learning segmentation of gadolinium-enhancing lesions in multiple sclerosis. Mult Scler 27(4):519–527 10.1177/1352458520921364 10.1177/1352458520921364 PMC7680286 32442043

[14] Anastasopoulos C, Reisert M, Kellner E (2017) Nora Imaging: a web-based platform for medical imaging. Neuropediatrics 48:P26

[15] Tustison NJ et al (2010) N4ITK: improved N3 bias correction. IEEE Trans Med Imaging 29:1310–1320 20378467 10.1109/TMI.2010.2046908 PMC3071855

[16] Klein S, Staring M, Murphy K, Viergever MA, Pluim JPW, Elastix (2010) A toolbox for intensity-based medical image registration. IEEE Trans Med Imaging 29:196–205 10.1109/TMI.2009.2035616 19923044

[17] Andermatt S, Pezold S, Cattin P (2016) Multi-dimensional gated recurrent units for the segmentation of biomedical 3D-data. in Lecture Notes in Computer Science (including subseries Lecture Notes in Artificial Intelligence and Lecture Notes in Bioinformatics) vol. 10008 LNCS 142–151Springer Verlag

[18] Fischl B et al (2002) Whole brain segmentation: automated labeling of neuroanatomical structures in the human brain. Neuron 33:341–355 11832223 10.1016/s0896-6273(02)00569-x

[19] Ashburner J, Friston KJ (2011) Diffeomorphic registration using geodesic shooting and Gauss-Newton optimisation. NeuroImage 55:954–967 21216294 10.1016/j.neuroimage.2010.12.049 PMC3221052

[20] Huisman M, van Ginneken B, Harvey H (2024) The emperor has few clothes: a realistic appraisal of current AI in radiology. Eur Radiol 2024:1–3. 10.1007/S00330-024-10664-0 10.1007/s00330-024-10664-0 38451323

[21] van Leeuwen KG, de Rooij M, Schalekamp S, van Ginneken B, Rutten MJ (2024) C. M. Clinical use of artificial intelligence products for radiology in the Netherlands between 2020 and 2022. Eur Radiol 34:348–354 37515632 10.1007/s00330-023-09991-5 PMC10791748

